# Symmetric Logarithmic Derivative of Fermionic Gaussian States

**DOI:** 10.3390/e20070485

**Published:** 2018-06-22

**Authors:** Angelo Carollo, Bernardo Spagnolo, Davide Valenti

**Affiliations:** 1 Department of Physics and Chemistry, Group of Interdisciplinary Theoretical Physics, Palermo University and CNISM, Viale delle Scienze, Ed. 18, I-90128 Palermo, Italy; 2 Department of Radiophysics, Lobachevsky State University of Nizhni Novgorod, 23 Gagarin Avenue, Nizhni Novgorod 603950, Russia; 3 Istituto Nazionale di Fisica Nucleare, Sezione di Catania, Via S. Sofia 64, I-90123 Catania, Italy; 4 Istituto di Biomedicina ed Immunologia Molecolare (IBIM) “Alberto Monroy”, CNR, Via Ugo La Malfa 153, I-90146 Palermo, Italy

**Keywords:** quantum metrology, Fermionic Gaussian state, quantum geometric information

## Abstract

In this article, we derive a closed form expression for the symmetric logarithmic derivative of Fermionic Gaussian states. This provides a direct way of computing the quantum Fisher Information for Fermionic Gaussian states. Applications range from quantum Metrology with thermal states to non-equilibrium steady states with Fermionic many-body systems.

## 1. Introduction

Quantum metrology or quantum parameter estimation is the theory that studies the accuracy by which a physical parameter of a quantum system can be estimated through measurements and statistical inference. In many physical scenarios, quantities which are to be estimated may not be directly observable, either due to experimental limitations or on account of fundamental principles. When this is the case, one needs to infer the value of the variable after measurements on a given probe. This is essentially a parameter estimation problem whose solution may be found using methods from classical estimation theory [1] or, when quantum systems are involved, from its quantum counterpart [2]. Quantum metrology finds applications in a range of diverse fields, from fundamental physics, such as improving frequency and time standards [3,4,5], estimating parameters in quantum field theory [6,7], improving the accuracy of gravitational wave interferometry [8,9], to applied physics, such as thermometry [10,11], spectroscopy [12,13], imaging [14,15,16], magnetic field detection [17,18] navigation [19,20] and remote sensing [21,22]. Exploiting the quantum nature of physical systems provides a remarkable advantage in enhancing the accuracy of estimation problems, and exploring this possibility plays a pivotal role in the current swift development of quantum technology [23,24,25,26,27,28,29,30,31,32,33,34]. Simultaneous quantum estimation of multiple parameters provides better precision over individual estimation strategies with equivalent resources [35,36]. This fact has sparked interest in multiparameter quantum metrology in a variety of scenarios [35,36,37,38].

Recent advances in quantum metrology have shown that the accuracy in parameter estimation can be enhanced by employing peculiar quantum many-body state as a probe [34,39]. Conversely, quantum metrology tools may well be exploited in the characterisation of many-body systems. Noteworthy instances of many-body quantum systems are those experiencing quantum phase transitions. Indeed, quantum parameter estimation, with its intimate relation with geometric information, provides a novel and promising approach in the characterisation of equilibrium [40,41,42,43,44,45,46,47,48,49] and out-of-equilibrium quantum critical phenomena [50,51,52,53,54].

Fermionic Gaussian states play a major role in the derivation of exact and approximate solution of many-body problems of Fermionic and spin systems. Deriving closed form expressions of quantities involved in parameter estimation problems for many-body quantum systems is a major challenge. This work addresses this task in the special, yet relevant, case of arbitrary Fermionic Gaussian states.

The solution of a parameter estimation problem amounts to find an estimator, i.e., a mapping $\hat{\lambda }=\hat{\lambda }\left(x_{1},x_{2},\ldots \right)$ from the set χ of measurement outcomes into the space of parameters λ∈M. Optimal estimators in classical estimation theory are those saturating the Cramer-Rao (CR) inequality,

(1) $${\mathrm{Cov}}_{\lambda }\left[\hat{\lambda }\right]\geq J^{c}{\left(\lambda \right)}^{-1},$$

which poses a lower bound on the mean square error ${\mathrm{Cov}}_{\lambda }{\left[\hat{\lambda }\right]}_{\mu \nu }=E_{\lambda }\left[{\left(\hat{\lambda }-\lambda \right)}_{\mu }{\left(\hat{\lambda }-\lambda \right)}_{\nu }\right]$ in terms of the Fisher information (FI)

(2) $$J_{\mu \nu }^{c}\left(\lambda \right)=\int_{\chi }d\hat{\lambda }\left(x\right)\;p\left(\hat{\lambda }|\lambda \right)\partial_{\mu }\log p\left(\hat{\lambda }|\lambda \right)\partial_{\nu }\log p\left(\hat{\lambda }|\lambda \right)\;.$$

For unbiased estimators, the mean square error is equal to the covariance matrix ${\mathrm{Cov}}_{\lambda }{\left[\hat{\lambda }\right]}_{\mu \nu }=E_{\lambda }\left[{\hat{\lambda }}_{\mu }{\hat{\lambda }}_{\nu }\right]-E_{\lambda }\left[{\hat{\lambda }}_{\mu }\right]E_{\lambda }\left[{\hat{\lambda }}_{\nu }\right]\;.$ The expression (1) should be understood as a matrix inequality. In general, one writes

$$\mathrm{tr}\left(W{\mathrm{Cov}}_{\lambda }\left[\hat{\lambda }\right]\right)\geq \mathrm{tr}\left(WJ^{c}{\left(\lambda \right)}^{-1}\right),$$

where *W* is a given positive definite cost matrix, which allows the uncertainty cost of different parameters to be weighed unevenly.

In the classical estimation problem, both in the single parameter case, and in the multiparameter one, the bound is saturable in the limit of an infinite number of repetitions of an experiment using the maximum likelihood estimator [55]. However, an interesting difference between multiparameter and single parameter metrology arises due to the correlation between parameters. Indeed, it may well happen that the resulting Fisher information matrix is non-diagonal. This means that the estimators for the parameters will not be independent. In a separate scheme in which all parameters except the $\lambda_{\mu }$ are perfectly known, the single parameter CR bound implies that the uncertainty of estimating $\lambda_{\mu }$ is lower bounded by $\mathrm{Var}\left(\hat{\lambda }\right)\geq 1/J_{\mu \mu }^{c}$. On the other hand, in the simultaneous scenario in which all parameters are estimated at the same time, one finds $\mathrm{Var}\left(\hat{\lambda }\right)\geq {\left(J^{c}{\left(\lambda \right)}^{-1}\right)}_{\mu \mu }$. From basic algebra of positive-definite matrices, we have that ${\left(J^{c}{\left(\lambda \right)}^{-1}\right)}_{\mu \mu }\geq 1/J^{c}{\left(\lambda \right)}_{\mu \mu }$, with equality holding only in the case when all off-diagonal elements vanish. Since asymptotically the CR bound is saturable, it implies that the equivalence between the simultaneous and separate scheme in the limit of a large number of experiment repetitions can only hold if *F* is a diagonal matrix, and hence there are no statistical correlations between the estimators [56].

Clearly, for any real positive definite matrix, one can perform an orthogonal rotation to a new basis in which the matrix is diagonal. This simply means that there are always linear combinations of the parameters for which the diagonality conditions hold. This choice should be, however, contrasted with the physical opportunity of performing such a rotation, as the choice of the parameters we are interested in may arise as a result of physical considerations and in this sense determine a preference in a specific basis.

While the fundamental objects in classical Fisher information are parameter-dependent probability-distribution of the data, the fundamental objects involved in the quantum estimation problem are the density matrices ρ(λ) labelled by λ∈M. In the quantum scenario, we therefore face the additional challenge of determining the optimal measurement for extracting most of the information on the parameters of interest from the quantum states. In the single parameter case, the situation is relatively simple. Maximization of the classical Fisher information over all quantum measurements yields the quantity referred to as the quantum Fisher information (QFI). The key object involved in the calculation of the QFI is the so-called *symmetric logarithmic derivative* (SLD), *L*, which is implicitly defined as the Hermitian operator satisfying the equation

(3) $$\frac{d\rho }{d\lambda }=\frac{1}{2}\left(\rho L+L\rho \right).\;$$

The QFI can be calculated using the formula:

(4) $$J=\mathrm{Tr}(\rho L^{2}).$$

One can always choose the projective measurement in the eigenbasis of the SLD that yields FI equal to the QFI. Hence, the QFI determines the ultimate achievable precision of estimating the parameter on density matrices ρ(λ) in the asymptotic limit of an infinite number of experiment repetitions. In a multiparameter scenario, a direct generalization of single parameter CR bound leads to the multiparameter quantum Cramer-Rao (QCR) bound [2,26,57], which reads

(5) $$\mathrm{tr}\left(W\mathrm{Cov}\left(\hat{\lambda }\right)\right)\geq \mathrm{tr}\left(WJ^{-1}\right),$$

where

(6) $$J_{\mu \nu }=\frac{1}{2}\mathrm{Tr}\rho \left\{L_{\mu },L_{\nu }\right\}$$

is the quantum Fisher information matrix (QFIM), *W* is the cost matrix, and $L_{\mu }$ is the SLD implicitly defined by Equation (3), with ρ derived with respect to the parameter $\lambda_{\mu }$.

Unlike the single parameter case, in the multiparameter scenario, the QCR bound cannot always be saturated. Intuitively, this is due to the incompatibility of the optimal measurements for different parameters. A sufficient condition for the saturation is indeed $[L_{\mu },L_{\nu }]=0$, which is however not a necessary condition. Within the comprehensive framework of quantum local asymptotic normality (QLAN) [58,59,60,61], a necessary and sufficient condition for the saturation of the multiparameter QCR bound is given by [62]

(7) $$U_{\mu \nu }=-\frac{i}{4}\mathrm{Tr}\rho \left[L_{\mu },L_{\nu }\right]=0\;\forall \mu ,\nu ,$$

and it is known as *compatibility condition* [62]. In the context of quantum information geometry, and quantum holonomies of mixed states, $U_{\mu \nu }$ is known as mean Uhlmann curvature [53].

In this paper, we derive a closed form expression of the SLD of Fermionic Gaussian states, which are of fundamental importance in the analysis of steady-states of both equilibrium and non-equilibrium quantum many-body systems, and their applications to quantum metrology.

The paper is organised as follows. In the next section, we shortly review the main properties of Fermionic Gaussian states. In Section 3, an explicit form for the calculation of the SLD is derived. In the last section, the conclusions are drawn.

## 2. Fermionic Gaussian State

We review here the main properties of Fermionic Gaussian states (FGSs). Let us consider a system of *n* fermionic particles described by creation and annihilation operators $c_{j}^{†}$ and $c_{j}$. These operators obey the canonical anticommutation relations,

(8) $$\left\{c_{j},c_{k}\right\}=0\;\left\{c_{j},c_{k}^{†}\right\}=\delta_{jk}\;.$$

Let us define the Hermitian Majorana operators as

(9) $$\omega_{2j-1}:=c_{j}+c_{j}^{†}\;,\;\omega_{2j}:=i\left(c_{j}-c_{j}^{†}\right)\;,$$

which are generators of a Clifford algebra, and satisfy the following anti-commutation relations

(10) $$\left\{\omega_{j},\omega_{k}\right\}=2\delta_{jk}\;.$$

Fermionic Gaussian states are defined as states that can be expressed as

(11) $$\rho =\frac{e^{-\frac{i}{4}\omega^{T}\Omega \omega }}{Z}\;,\;Z:=\mathrm{Tr}\left[e^{-\frac{i}{4}\omega^{T}\Omega \omega }\right],$$

where Ω is a 2n×2n real antisymmetric matrix and $\omega :={\left(\omega_{1}\cdots \omega_{2n}\right)}^{T}$ is a 2n-dimensional array of Majorana Fermions. As any antisymmetric real matrix, Ω can be cast in the following canonical form by an orthogonal matrix *Q*, i.e.,

(12) $$\begin{array}{ccc} \Omega & =Q^{T}\;\overset{n}{\underset{k=1}{⨁}}\left(\begin{array}{cc} 0 & \Omega_{k}\\ -\Omega_{k} & 0 \end{array}\right)\;Q & Q^{T}=Q^{-1}\;, \end{array}$$

where $\pm i\Omega_{k}$ are Ω’s eigenvalues. Let

(13) $$z={\left(z_{1},\cdots ,z_{2n}\right)}^{T}:=Q\omega$$

be the vector of Majorana fermions in the eigenmode representation. Hence,

(14) $$\begin{array}{cc} \rho & =\frac{1}{Z}\prod_{k}\left[\cosh\left(\frac{\Omega_{k}}{2}\right)-i\sinh\left(\frac{\Omega_{k}}{2}\right)\;z_{2k-1}z_{2k}\right]\;, \end{array}$$

(15) $$\begin{array}{cc} Z & =\prod_{k}2\cosh\left(\frac{\Omega_{k}}{2}\right)\;. \end{array}$$

Gaussian states are completely specified by the two-point correlation matrix

(16) $$\Gamma_{jk}:=1/2\mathrm{Tr}\left(\rho \left[\omega_{j},\omega_{k}\right]\right)\;,\;\Gamma =\Gamma^{†}=-\Gamma^{T}\;,$$

which is an imaginary antisymmetric matrix. Let’s recall some basic properties of the correlation function. As for any Fermionic State, all odd-order correlation functions of FGSs are zero, due to the parity super-selection rule. In FGSs, all even-order correlations, higher than two, can be obtained from Γ by Wick’s theorem [63], i.e.,

(17) $$\mathrm{Tr}\left(\rho \omega_{k_{1}}\omega_{k_{2}}\ldots \omega_{k_{2p}}\right)=\mathrm{Pf}\left(\Gamma_{k_{1}k_{2}\cdots k_{2p}}\right),\;1\leq k_{1}<\ldots <k_{2p}\leq 2n$$

and $\Gamma_{k_{1}k_{2}\cdots k_{2p}}$ is the corresponding 2p×2p submatrix of Γ. $\mathrm{Pf}{\left(\Gamma_{k_{1}k_{2}\cdots k_{2p}}\right)}^{2}=\det\left(\Gamma_{k_{1}k_{2}\cdots k_{2p}}\right)$ is the Pfaffian. An especially useful case is the four-point correlation function

(18) $$\mathrm{Tr}\left(\rho \omega_{j}\omega_{k}\omega_{l}\omega_{m}\right)=a_{jk}a_{lm}-a_{jl}a_{km}+a_{jm}a_{kl},$$

where $a_{jk}:=\Gamma_{jk}+\delta_{jk}$. As

(19) $$\Gamma_{jk}=\frac{2i}{Z}\frac{\partial Z}{\partial \Omega_{jk}},$$

one can show that

(20) $$\begin{array}{c} \Gamma =\tanh\left(i\frac{\Omega }{2}\right)\;. \end{array}$$

The correlation matrix is diagonal in the same basis of Ω and its eigenvalues read $\gamma_{k}=\tanh\left(\Omega_{k}/2\right)$. Hence

(21) $$\begin{array}{cc} \rho & =\prod_{k=1}^{n}\frac{1-i|\gamma_{k}|\;z_{2k-1}z_{2k}}{2}\;, \end{array}$$

where $|\gamma_{k}|\leq 1$. Hence, the Gaussian fermionic state can be factorised into a tensor product $\rho ={⨂}_{k}\rho_{k}$ of density matrices of the eigenmodes $\rho_{k}:=\frac{1-i|\gamma_{k}|\;z_{2k-1}z_{2k}}{2}$. Note that, for $\gamma_{k}=\pm 1$, one has $\Omega_{k}=\pm \infty$, making the definition (11) of Gaussian states not well defined, unlike Equation (21), showing that the latter offer an appropriate parameterisation even in those extremal points. Notice that $|\gamma_{k}|=1$ corresponds to a fermionic mode ${\tilde{c}}_{k}=1/2\left(z_{2k-1}+z_{2k}\right)$ being in a pure state, as it is clear from the following explicit expression for the purity of the states $\rho_{k}$:

(22) $$\begin{array}{c} \mathrm{Tr}\left[\rho_{k}^{2}\right]=\frac{\det{\left[2\cosh\left(\;\Omega_{k}\right)\right]}^{\frac{1}{2}}}{\det\left[2\cosh\left(\frac{\Omega_{k}}{2}\right)\right]}, \end{array}$$

(23) $$\begin{array}{c} \mathrm{Tr}\left[\rho^{2}\right]=\frac{\det{\left[2\cosh\left(i\;\Omega \right)\right]}^{\frac{1}{2}}}{\det\left[2\cosh\left(i\frac{\Omega }{2}\right)\right]}=\sqrt{\det\left(\frac{1+\Gamma^{2}}{2}\right)}\;. \end{array}$$

## 3. Symmetric Logarithmic, Derivative of Fermionic Gaussian States

We will derive here an explicit formula for the calculation of the SLD for Fermionic Gaussian states. To this end, we review a useful expression adapted from [64] needed for the derivation of the symmetric logarithmic derivative of density matrices in the exponential form

(24) $$\rho =e^{D(\lambda )}.$$

Clearly, a Gaussian Fermionic state can be expressed in the exponential form (24) by identifying

(25) $$D=-\frac{i}{4}\omega^{T}\cdot \Omega \cdot \omega -1\;\;1\ln Z.$$

Notice that the above parameterisation is well defined only in the case of full-rank density matrices. As usual, for the case of extremal conditions $|\gamma_{k}|=1$, an eigenvalue of the correlation function should be carried out as a limiting procedure.

The starting point is the expression derived in equation (2.1) of [65] for derivative of density operators

(26) $$\begin{array}{c} \dot{\rho }=\int_{0}^{1}e^{sD}\;\dot{D}\;e^{(1-s)D}\;ds\;, \end{array}$$

where dots represent derivatives with respect to a parameter λ. One can use the nested- commutator relation

(27) $$\begin{array}{cc} e^{D}Ae^{-D} & =A+\left[D,A\right]+\frac{1}{2!}\;\left[D,[D,A]\;\right]+\cdots \\ & =\sum_{n=0}^{\infty }\frac{1}{n!}\;C^{n}\left(A\right)=e^{C}\left(A\right)\;, \end{array}$$

where $C^{n}\left(A\right)$, a linear operation on *A*, denotes the *n*th-order nested commutator $\left[\;D\;,\;\;\ldots \;,[\;D\;,\;A\;]\;\right]$, with $C^{0}\left(A\right)=A$. Applying this relation to the expression (26) leads to

(28) $$\begin{array}{c} \begin{array}{cc} \dot{\rho }\rho^{-1} & =\dot{D}+\frac{1}{2!}\;\left[\;D\;,\;\dot{D}\;\right]+\frac{1}{3!}\;\left[\;D\;,\;[\;D\;,\;\dot{D}\;]\;\right]+\cdots \\ & =\sum_{n=0}^{\infty }\frac{1}{(n+1)!}\;C^{n}\left(\dot{D}\right)=h\left(C\right)\left(\dot{D}\right)\;, \end{array} \end{array}$$

where *h* is the generating function of the expansion coefficients in Equation (28),

(29) $$\begin{array}{c} h\left(t\right)=1+\frac{t}{2!}+\frac{t^{2}}{3!}+\cdots =\frac{e^{t}-1}{t}\;. \end{array}$$

Using the definition of symmetric logarithmic derivative, i.e.,

(30) $$\dot{\rho }=\frac{1}{2}\left(L\rho +\rho L\right)\;,$$

and that of density matrix in exponential form (24), one gets

(31) $$\begin{array}{c} \begin{array}{cc} \dot{\rho }\;\rho^{-1} & =\frac{1}{2}\left(L+e^{D}Le^{-D}\right)\\ & =\frac{1}{2}\left(L+\sum_{n=0}^{\infty }\frac{1}{n!}\;C^{n}\left(L\right)\right)=r\left(C\right)\left(L\right)\;, \end{array} \end{array}$$

where the generating function is $r\left(t\right)=\left(e^{t}+1\right)/2$. Suppose that the SLD adopts the form,

(32) $$\begin{array}{cc} L & =\sum_{n=0}^{\infty }f_{n}\;C^{n}\left(\dot{D}\right)=f\left(C\right)\left(\dot{D}\right)\;, \end{array}$$

with the generating function

(33) $$\begin{array}{c} f\left(t\right)=f_{0}+f_{1}t+f_{2}t^{2}+\cdots \end{array}$$

to be determined. Plugging Equation (32) into Equation (31) yields

(34) $$\begin{array}{c} \dot{\rho }\;\rho^{-1}=r\left(C\right)\left[f\left(C\right)\left(\dot{D}\right)\right]=r\circ f\left(C\right)\left(\dot{D}\right)=r\cdot f\left(C\right)\left(\dot{D}\right)\;, \end{array}$$

where the identity r∘f=r·f, between the combination, r∘f, of the two functions and their simple product, r·f, arises from $C^{n}\left(C^{m}\left(A\right)\right)=C^{n+m}\left(A\right)$. Comparing Equation (34) with Equation (28) leads to the following relation between generating functions,

(35) $$\begin{array}{c} f\left(t\right)=\frac{h(t)}{r(t)}=\frac{\tanh(t/2)}{t/2}=\sum_{n=0}^{\infty }\frac{4\;\left(4^{n+1}-1\right)B_{2n+2}}{(2n+2)!}\;t^{2n}\;, \end{array}$$

where $B_{2n+2}$ is the (2n+2)th Bernoulli number. Comparing Equations (33) with (35), we have

(36) $$\begin{array}{c} f_{n}=\left\{\begin{array}{cc} \frac{4\;\left(4^{n/2+1}-1\right)B_{n+2}}{(n+2)!}\;, & \mathrm{for}\;\mathrm{even}\;n\;,\\ 0\;, & \mathrm{for}\;\mathrm{odd}\;n\;. \end{array}\right. \end{array}$$

The vanishing of the odd-order of $f_{n}$s is a consequence of the Hermiticity of *L*, which makes f(t) an even function. From the definition (25) of *D*, one straightforwardly finds

(37) $$\dot{D}=-\frac{1}{2}\;\;\omega^{T}\cdot \;\dot{\Omega }\cdot \omega -\frac{\dot{Z}}{Z}1\;\;1\;,$$

which shows that $\dot{D}$ is itself a quadratic function of the Majorana Fermion operators, where $\dot{\Omega }$ is an antisymmetric real matrix given by

(38) $$\begin{array}{cc} \dot{\Omega } & =Q^{T}\;\overset{n}{\underset{k=1}{⨁}}\left(\begin{array}{cc} 0 & {\dot{\Omega }}_{k}\\ -{\dot{\Omega }}_{k} & 0 \end{array}\right)Q+i\left[R,\Omega \right]\;, \end{array}$$

with $R:=iQ^{T}\dot{Q}$. Therefore, for a Gaussian Fermionic state, the operator $\dot{D}$ can be written in a canonical form in terms of the Majorana Fermions of its own eigenmodes, as

(39) $$\dot{D}=-\frac{i}{4}\sum_{k}{\tilde{\Omega }}_{k}\left[{\tilde{z}}_{2k-1},{\tilde{z}}_{2k}\right]-\frac{\dot{Z}}{Z}1\;\;1=\sum_{k}{\tilde{\Omega }}_{k}\left({\tilde{c}}_{k}^{†}{\tilde{c}}_{k}-\frac{1}{2}\right)-\frac{\dot{Z}}{Z}1\;\;1\;,$$

where ${\tilde{c}}_{k}:=\frac{1}{2}\left({\tilde{z}}_{2k-1}+i{\tilde{z}}_{2k}\right)$, ${\tilde{c}}_{k}^{†}:=\frac{1}{2}\left({\tilde{z}}_{2k-1}-i{\tilde{z}}_{2k}\right)$ are the ordinary annihilation and creation operators of the eigenmodes of $\dot{D}$, and ${\tilde{\Omega }}_{k}$ the corresponding eigenvalues. It is straightforward to derive the commutation relations between $\dot{D}$ and Fermionic operators,

(40) $$\begin{array}{c} \left[\;\dot{D}\;,\;{\tilde{c}}_{k}\;\right]=-{\tilde{\Omega }}_{k}{\tilde{c}}_{k}\;,\;\left[\;\dot{D}\;,\;{\tilde{c}}_{k}^{†}\;\right]=\;{\tilde{\Omega }}_{k}{\tilde{c}}_{k}^{†}\;, \end{array}$$

and for quadratic operators, also

(41) $$\begin{array}{c} \left[\;\dot{D}\;,\;{\tilde{c}}_{j}^{†}{\tilde{c}}_{k}\;\right]=\left({\tilde{\Omega }}_{j}-{\tilde{\Omega }}_{k}\right){\tilde{c}}_{j}^{†}\;{\tilde{c}}_{k},\;\left[\;\dot{D}\;,\;{\tilde{c}}_{j}^{†}{\tilde{c}}_{k}^{†}\;\right]=\left({\tilde{\Omega }}_{j}+{\tilde{\Omega }}_{k}\right){\tilde{c}}_{j}^{†}{\tilde{c}}_{k}^{†}. \end{array}$$

Consequently, one finds

(42) $$\begin{array}{c} f\left(C\right)\;\left({\tilde{c}}_{j}^{†}{\tilde{c}}_{k}\right)=f\left({\tilde{\Omega }}_{j}-{\tilde{\Omega }}_{k}\right)\;{\tilde{c}}_{j}^{†}{\tilde{c}}_{k}\;, \end{array}$$

(43) $$\begin{array}{c} f\left(C\right)\;\left({\tilde{c}}_{j}^{†}{\tilde{c}}_{k}^{†}\right)=f\left({\tilde{\Omega }}_{j}+{\tilde{\Omega }}_{k}\right)\;{\tilde{c}}_{j}^{†}{\tilde{c}}_{k}^{†}\;. \end{array}$$

The above expression, plugged into formula (32), shows that *L* is at most quadratic in the Fermionic operators.

Due to the quadratic dependence of *L* on the Fermionic operator, clearly *L* can be expressed as a quadratic polynomial in the Majorana Fermions in the following form

(44) $$L_{}=:\frac{1}{2}\omega^{T}\cdot K\cdot \omega +\zeta_{}^{T}\cdot \omega +\eta ,$$

where $K:={\left\{K_{jk}\right\}}_{jk=1}^{2n}$ is 2n×2n Hermitian anti-symmetric matrix, $\zeta :={\left\{\zeta^{k}\right\}}_{k=1}^{2n}$ is a 2n real vector, and η is a real number. Note that any odd-order correlation function for a Gaussian Fermionic state vanishes identically, then

(45) $$\langle \omega_{k}\rangle =\mathrm{Tr}\left(\rho \omega_{k}\right)=0\;\forall k=1\cdots 2n\;.$$

By differentiating the above equation, one readily shows that the linear term in (44) is identically zero

$$0=\frac{d}{d\lambda }\mathrm{Tr}\left(\rho \omega_{k}\right)=\mathrm{Tr}\left(\omega_{k}\dot{\rho }\right)=\frac{1}{2}\mathrm{Tr}\left(\omega_{k}\left\{L,\rho \right\}\right)=\mathrm{Tr}\left(\rho \left\{\zeta^{T}\omega ,\omega_{k}\right\}\right)=\zeta^{k}\;,$$

where $\zeta^{k}$ is the *k*-th component of ζ, and, in the fourth equality, one takes into account that the third order correlations vanish. The quantity η can be determined from the trace preserving condition, i.e.,

(46) $$0=\frac{d}{d\lambda }\;\mathrm{Tr}\;\rho =\mathrm{Tr}\left(\dot{\rho }\right)=\mathrm{Tr}\left(\rho L\right)\;,$$

which, after plugging in Equation (44), leads to

(47) $$\eta =-\frac{1}{2}\mathrm{Tr}\left(\rho \omega^{T}K\omega \right)=\frac{1}{2}\mathrm{Tr}\left(K_{}\;\Gamma \right).$$

In order to determine *K*, let’s take the differential of $\Gamma_{jk}=1/2\mathrm{Tr}\left(\rho \left[\omega_{j},\omega_{k}\right]\right)$, then

(48) $$\begin{array}{c} \begin{array}{cc} {\dot{\Gamma }}_{jk}=\frac{1}{2}\mathrm{Tr}\left(\dot{\rho }\left[\omega_{j},\omega_{k}\right]\right) & =\frac{1}{4}\mathrm{Tr}\left(\left\{\rho ,L_{}\right\}\left[\omega_{j},\omega_{k}\right]\right)\\ & =\frac{1}{8}\mathrm{Tr}\left(\left\{\rho ,\omega^{T}K_{}\omega \right\}\left[\omega_{j},\omega_{k}\right]\right)+\frac{\eta_{}}{4}\mathrm{Tr}\left(\rho \left[\omega_{j},\omega_{k}\right]\right)\\ & =\frac{1}{8}\sum_{lm}K_{}^{lm}\mathrm{Tr}\left(\left\{\rho ,\left[\omega_{l},\omega_{m}\right]\right\}\left[\omega_{j},\omega_{k}\right]\right)+\frac{\eta_{}}{2}\Gamma_{jk}\\ & ={\left(\Gamma K_{}\Gamma -K_{}\right)}_{jk}+\frac{1}{2}\left[\eta -\frac{1}{2}\mathrm{Tr}\left(K_{}\;\Gamma \right)\right]\Gamma_{jk}, \end{array} \end{array}$$

where the last equality is obtained with the help of Equation (18) and using the antisymmetry of Γ and *K* under the exchange of *j* and *k*. Finally, according to Equation (47), the last term vanishes and we obtain the following (discrete time) Lyapunov equation

(49) $$\dot{\Gamma }=\Gamma K_{}\Gamma -K_{}.$$

The above equation can be formally solved by

(50) $$K_{}={\left({\mathrm{Ad}}_{\Gamma }-1\;\;1\right)}^{-1}\left(\dot{\Gamma }\right),$$

where ${\mathrm{Ad}}_{\Gamma }\left(X\right):=\Gamma X\Gamma^{†}$ is the adjoint action. In the eigenbasis of Γ, (i.e., $\Gamma |j\rangle =\gamma_{j}|j\rangle$), it reads

(51) $$\langle j|K_{}|k\rangle ={\left(K_{}\right)}_{jk}=\frac{{\left(\dot{\Gamma }\right)}_{jk}}{\gamma_{j}\gamma_{k}-1}=-\frac{{\dot{\Omega }}_{k}}{2}\delta_{jk}+\tanh\frac{\Omega_{j}-\Omega_{k}}{2}\langle j|\dot{k}\rangle ,$$

where, in the second equality, we made use of the relation $\gamma_{k}=\tanh\left(\Omega_{k}/2\right)$, which yields the following diagonal ${\left(\dot{\Gamma }\right)}_{jj}=\left(1-\gamma_{j}^{2}\right){\dot{\Omega }}_{j}$ and off-diagonal ${\left(\dot{\Gamma }\right)}_{jk}=\left(\gamma_{k}-\gamma_{j}\right)\langle j|\dot{k}\rangle$ terms. This expression is well defined everywhere except for $\gamma_{j}=\gamma_{k}=\pm 1$, where the Gaussian state ρ becomes singular (i.e., it is not full rank). In this condition, the expression (51) for the SLD *L* may become singular. Nevertheless, the boundness of the function $|\tanh\frac{\Omega_{j}-\Omega_{k}}{2}|\leq 1$ in (51) shows that such a singularity is relatively benign. One can show that the condition $\gamma_{j}=\gamma_{k}=\pm 1$ produces, at most, removable singularities in the quantum Fisher Information Matrix (cf. [66]). This allows the quantum Fisher information matrix to be extended by continuity from the set of full-rank density matrices to the subset with $\gamma_{j}=\gamma_{k}=\pm 1$.

## 4. Conclusions

In this work, we derived a general expression for the symmetric logarithmic derivative of an arbitrary Fermionic Gaussian state. We obtained a compact expression in terms of a correlation matrix of a FGS, which allows for the calculation of the quantum Fisher information. This provides a way of assessing the ultimate precision of parameter estimation problems in many-body systems involving Fermionic Gaussian states, through the quantum Cramer-Rao bound. Moreover, the expression of the SLD allows for the explicit derivation of the eigenbasis associated with the optimal quantum measurement to be performed for the estimation of a parameter of interest. The generality of the method also offers a way of evaluating the so-called *compatibility condition* in multiparameter quantum estimation problems [62]. Indeed, due to the quantum nature of the underlying probe, the multiparameter estimation problem may not saturate the multiparameter quantum Cramer-Rao bound. Unlike classical estimation problems, the non-commutativity of the observables involved in the optimal quantum measurements associated with independent parameters may prevent the quantum CR bound from being saturated [29]. An explicit quantitative condition [62] for such a compatibility can be easily derived, once the formula for the SLD is given. The general framework presented provides a way of easily assessing the above-mentioned quantities. Moreover, the explicit expression of the SLD, analogous to Bosonic Gaussian estimation problems [31], can be exploited, in combination with efficient numerical algorithms, to find an optimal Fermionic Gaussian probe that minimises the overall multiparameter estimation problem [67].
